# Cytologic features of nipple aspirate fluid using an automated non-invasive collection device: a prospective observational study

**DOI:** 10.1186/1472-6874-5-10

**Published:** 2005-08-03

**Authors:** Kerry AS Proctor, Leslie R Rowe, Joel S Bentz

**Affiliations:** 1 Department of Pathology, University of Utah, Salt Lake City, Utah, USA; 2 Institute for Clinical and Experimental Pathology, Associated Regional and University Pathologists (ARUP) Laboratories Inc., Salt Lake City, Utah, USA

## Abstract

**Background:**

Detection of cytologic atypia in nipple aspirate fluid (NAF) has been shown to be a predictor of risk for development of breast carcinoma. Manual collection of NAF for cytologic evaluation varies widely in terms of efficacy, ease of use, and patient acceptance. We investigated a new automated device for the non-invasive collection of NAF in the office setting.

**Methods:**

A multi-center prospective observational clinical trial involving asymptomatic women designed to assess fluid production, adequacy, safety and patient acceptance of the HALO NAF Collection System (NeoMatrix, Irvine, CA). Cytologic evaluation of all NAF samples was performed using previously described classification categories.

**Results:**

500 healthy women were successfully enrolled. Thirty-eight percent (190/500) produced fluid and 187 were available for cytologic analysis. Cytologic classification of fluid producers showed 50% (93/187) Category 0 (insufficient cellular material), 38% (71/187) Category I (benign non-hyperplastic ductal epithelial cells), 10% (18/187) Category II (benign hyperplastic ductal epithelial cells), 3% (5/187) Category III (atypical ductal epithelial cells) and none were Category IV (unequivocal malignancy). Overall, 19% of the subjects produced NAF with adequate cellularity and 1% were found to have cytologic atypia.

**Conclusion:**

The HALO system is a simple, safe, rapid, automated method for standardized collection of NAF which is acceptable to patients. Cytologic assessment of HALO-collected NAF showed the ability to detect benign and pre-neoplastic ductal epithelial cells from asymptomatic volunteers.

## Background

The majority of breast cancers originate in the epithelium lining the milk ducts. It is believed that most breast cancers are slow growing and progress from precancerous cells, which have cellular and nuclear changes that can be identified microscopically. Finding microscopic evidence of ductal epithelial atypia/atypical ductal hyperplasia (ADH) has been shown in previous epidemiologic studies to be a predictor of future breast cancer development in an individual woman. [1-10] This increased risk has been identified using random peri-areolar fine needle aspiration (FNA), tissue biopsy or nipple secretion samples for assessment of cytologic atypia.

Nipple fluid or secretions, usually aspirated from the breast ducts, is a protein rich material termed nipple aspirate fluid (NAF) which can be microscopically examined for the presence of atypical ductal epithelial cells. Nipple fluid can be obtained from many women, with reports of NAF production ranging from 25% [11] to more than 95% [12] of women. There are a variety of factors associated with the ability to produce nipple fluid, particularly intrinsic breast characteristics [13]. Nipple fluid acquisition methods are various, including manual breast compression, either followed by manual breast pump or syringe-type device with suction, sometimes repeated up to 10 minutes on each breast.

Breast cancer risk assessment using breast fluid cytology has been suggested to have a role in risk stratification and clinical decision making for women who are at high risk for breast cancer development. Ductal lavage is for clinical use in high-risk women and involves identification and cannulation of one or more fluid-yielding duct(s) then rinsing each with saline, collecting and analyzing the lavaged fluid. The finding of atypical cells could potentially influence a woman's decision for more aggressive surveillance or chemoprevention. Women with atypical ductal hyperplasia in the Breast Cancer Prevention Trial showed an 86% risk reduction with tamoxifen chemoprevention [14].

We analyzed cytologic features of samples obtained during a pilot study using a new suction-based automated mechanical device for the non-invasive collection of NAF in the office setting. This article reports the results from a multi-center prospective observational clinical trial involving asymptomatic women designed to evaluate fluid production, adequacy, safety, patient acceptance and ability to detect atypical breast epithelial cells.

## Methods

### Study sponsor and study design

The study sponsor was Neomatrix, LLC (Irvine, Ca). The study design and execution was the responsibility of the study sponsor. The author's institution (ARUP Laboratories) provided contracted pathology services for the study sponsor, on a usual and customary fee-for-service basis. Study administration expenses at the parent institution (University of Utah) were paid by the study sponsor. No direct compensation was made to the manuscript authors.

The study was conducted over a one year period and no preparatory phase was incorporated. The participant enrollment sites were obstetric and gynecology clinic practices located in Avon, CT., Farmington, CT., and Baton Rouge, LA. There was a single set-up instructional visit that took less than one hour and was performed prior to initiation of the investigation. The only variation in collection rates was seen between centers. One site had a lower NAF collection rate initially. The HALO System at this site was evaluated and the equipment was found to be operating outside of its specified performance parameters. The equipment was replaced and the variability in NAF collection rates between centers was no longer observed. During the study, enrollment was stopped for approximately 6 months in order to make some minor design modifications to enhance equipment performance.

### Patient enrollment

The study population included only asymptomatic, non-pregnant, non-lactating women with no history of breast cancer, breast surgery (e.g. breast augmentation or breast reduction), or nipple piercing who were asked to volunteer as part of a prospective multi-center observational study.

Participants were required to be at least 18 years old and there was no upper age limit. Written informed consent was obtained from all subjects before enrollment in the study. The study protocol was approved for all participating collection centers by Biomedical Research Institute of America (San Diego, CA), an independent Institutional Review Board (Protocol PP-01) and the University of Utah (IRB #11588) Institutional Review Board. All rules and regulations concerning biomedical studies with human subjects were followed. A standardized questionnaire was completed for all participants that included medical history, current medications, family history, obstetric and gynecologic history, and breast health history including any previous biopsies and mammogram results. Gail model 5-year risk profiles were calculated for each woman over the age of 35 .

### Nipple aspiration procedure

**Figure 1:**
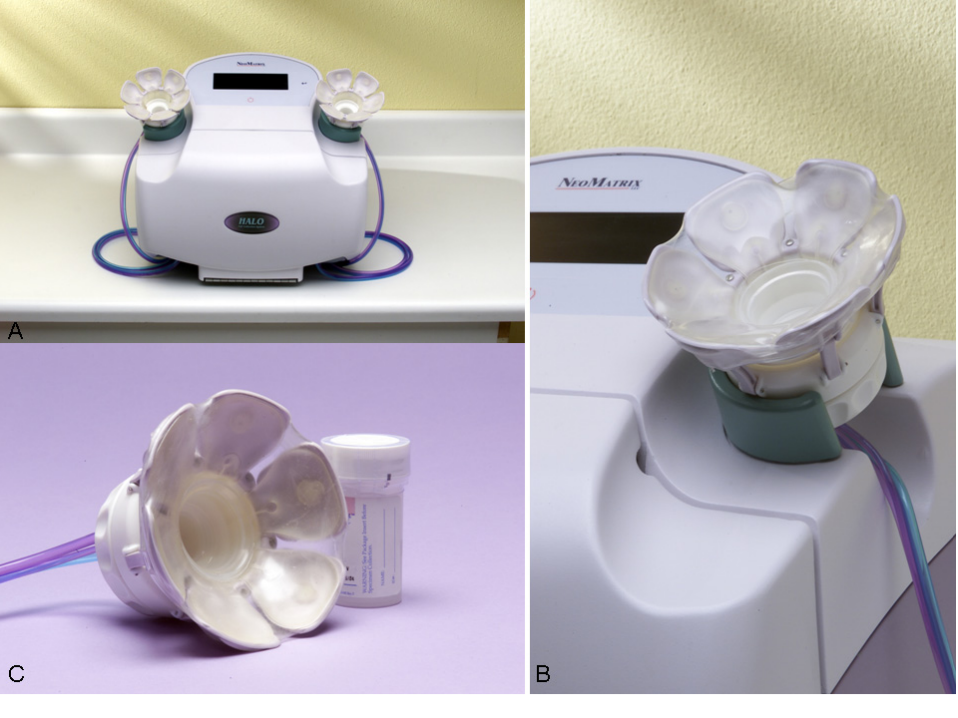
The HALO NAF collection system (photos courtesy of NeoMatrix, Irvine, CA). A. Control Console B. Adjustable Breast Cups with Fluid Reservoir Cassette C. Disposable Sample Collection Cups.

**Figure 2:**
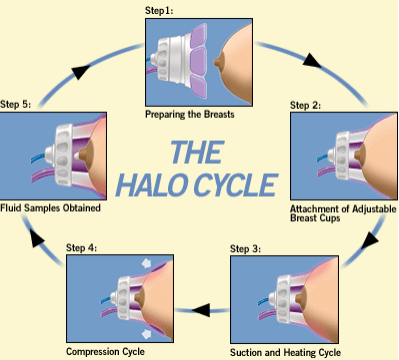
The HALO device applied to a breast. (Photos courtesy of NeoMatrix, Irvine, CA.) An alcohol wipe is used to cleanse the nipples (step 1); cups are placed on the woman's breasts and adjusted to fit (step 2); heat and suction are simultaneously applied via the breast cup petals (step 3); mild compression is initiated by the console (step 4); any sample of NAF obtained is transferred to a vial of cytology fixative and transported to the cytology lab (step 5).

After obtaining written informed consent and completing a study questionnaire, a clinical breast exam was performed. The subject was wearing a front-opening examination gown and seated in a comfortable position. Each breast was cleaned using an alcohol wipe. Abrasive cleanser was not used. The HALO NAF Collection System (NeoMatrix, Irvine, CA) (Fig 1) has adjustable breast cups with disposable sample collection cups which were placed simultaneously on each breast and manually adjusted to fit snugly around the nipple and areola. The application of topical anesthetic was not required. Occasionally, some breast tissue proximal to the areola was covered by the petals depending on the breast size. After both breast cups are secure, the START button is depressed to initiate the automatic NAF acquisition cycle (Fig. 2). The HALO console initiates the vacuum, providing a gentle suction (similar to that of a breast pump) on both breasts. Simultaneously with suction, heat is applied to the covered areas via circulating warm fluid within the Breast Cup petals. Towards the end of the cycle, the HALO system initiates mild compression of the Breast Cup petals to retrieve any fluid from the ducts. The entire cycle is 5 minutes in duration. The Console indicates when the NAF acquisition cycle has completed. Suction is gently automatically released from the breast cups.

Any collected NAF was transferred from the nipple or sample collection cup(s) to a vial of fixative (CytoLyt, Cytyc Corporation, Boxborough, MA) using a pipette if necessary. If fluid was obtained from either one or both breasts, all samples were combined into a single sample preservative vial. Only one attempt was made to obtain NAF in the five minute session, and if no NAF was produced by either breast, the participant was considered to be a non-producer.

### Sample processing and cytologic examination

All samples were shipped to a single reference laboratory (ARUP Laboratories, Inc., Salt Lake City, UT). Microscopic slides were prepared from the entire NAF sample using a Millipore filter technique (Millipore Corp., Billerica, MA), which was chosen due to the low cellularity of the specimens. The filter preps were stained with the modified Papanicolaou stain technique.

Each slide was reviewed by one of a group of three cytopathologists with experience examining breast cytologic specimens, including ductal lavage, and who were trained in the NAF cytologic categories prior to beginning the study. All difficult or borderline cases were resolved by reviewing cases at a multi-headed microscope. The slides were classified according to the most severe abnormality detected using one of five categories based upon increasing nuclear abnormality. The classification system used categories developed by King et al (Table 1). Category 0 was designated for unsatisfactory specimens, with insufficient material for complete evaluation, defined microscopically as having less than 10 ductal epithelial cells. Category I samples contained benign non-hyperplastic ductal epithelial cells. Category II was defined as ductal epithelial hyperplasia with cellular arrangements of cohesive clusters (greater than 10 – 50 cells) and minimal cellular changes including mild nuclear and cellular enlargement and occasional nucleoli but finely granular and evenly distributed chromatin. Category III, atypical hyperplasia, included cells with more distinct nuclear enlargement, increased nuclear to cytoplasmic ratios, coarsely granular chromatin with prominent chromocenters and irregular nuclear membranes with nuclear variation. Increased numbers of atypical single cells were also included in this group. Category IV was defined as unequivocally malignant cells.

**Table 1:** Nipple aspirate fluid cytology classification*

Classification	Characteristics	Interpretation
Unsatisfactory (Category 0)	<10 ductal epithelial cells.	Unsatisfactory specimen.
Benign (Category I)	Duct epithelial cells within normal limits. Foam cells. Apocrine metaplastic cells.	No malignant cells identified. Benign (non-hyperplastic) ductal epithelial cells present.
Hyperplasia (Category II)	Minimal changes including slight cell and nuclear enlargement. Chromatin remains finely granular and evenly distributed. Small and regular nucleoli sometimes present. Cell distribution predominately in groups and cohesive with >10–50 cells (papillary and apocrine subcategories).	No malignant cells identified. Benign hyperplastic ductal epithelial cells present.
Atypical Hyperplasia (Category III)	Moderate to severe abnormalities with distinct nuclear enlargement, increasing nuclear to cytoplasmic ratio, irregular nuclear borders, and nuclear variation. Coarsely granular chromatin. Prominent chromocenters. Cell distribution in groups with some papillary formations. Increased numbers of single atypical cells (apocrine type subcategory).	Atypical hyperplastic ductal epithelial cells present. Malignancy cannot be completely excluded.
Malignancy (Category IV)	Single cells and groups of cells with unequivocal nuclear features of cancer.	Malignant cells present derived from adenocarcinoma.

* King et al [4]

### Data collection and monitoring

Enrollment site investigators and study coordinators filled out case report forms recording relevant information about the subjects' medical history, eligibility, study procedures, adverse events, and any available follow-up care. All clinical sites were monitored and all study data, including final cytology results, where collected by the study sponsor, NeoMatrix.

### Patient acceptance and post-procedure survey

Adverse events were noted immediately after the procedure as well as at the four to eight week post-procedure survey. The participant was asked to rate her comfort level immediately following the procedure using a visual analog scale of 1 to 10, with one being most comfortable and 10 being least comfortable. Participants were also contacted four to eight weeks after the procedure to assess satisfaction with the procedure. Women with cytologic atypia or worse (Class III or IV) were referred to their regular physicians who determined the appropriate follow-up care. A standard protocol for following patients with atypia was not included as part of this investigation. This follow-up care may have included further assessment of the patient via imaging, biopsy in some cases, risk counseling, and increased surveillance.

### Statistical analysis

The result of NAF producers and non-producers were expressed as raw numbers for each demographic category. Comparison between two groups was performed using the χ^2 ^test of association. The difference between values was considered significant at *P *< 0.05.

## Results

### Enrollment demographics

Five hundred (n = 500) participants were successfully enrolled. Overall characteristics of the study participants are summarized in Table 2. The average age was 41.1 years (range 18–65). There was no significant difference between age and fluid production (p <= 1.0). One-hundred and ninety (38%) of the women were fluid producers. Thirty-eight percent (162/426) of women less than 55 years old were fluid producers, while 38% (28/74) of women aged 55 or older produced fluid. Eighty-nine percent (445/500) were Caucasian, 9% (47/500) were African American, 1% (7/500) were Hispanic and one participant was Asian. Forty-eight percent of nulliparous women were fluid producers whereas 36% of parous women produced NAF, which is not statistically significant for the group of pre-menopausal women (p <= 1.0), but is significant if all women are included (p < 0.05). Thirty-nine percent of Caucasians produced NAF while NAF was obtained from 31% of non-white subjects (p <= 1.0). Thirty-six percent of subjects with no 1^st ^degree family history produced NAF, 46% of subjects with one 1^st ^degree relative with breast cancer, and 75% of subjects who had more than one 1^st ^degree relative with breast cancer (p <= 0.10). Forty-two percent of women with a lactation history produced NAF while 34% of women who never lactated were fluid producers (p < 0.10). Overall, 14% had at least one first degree relative with cancer and 11% had a history of a previous breast biopsy. In summary, none of the differences between fluid producers and non-producers with regards to any of the listed demographics was statistically significant.

**Table 2:** Participant characteristics, demographics and fluid production status

	Overall, No. (%)	NAF Producers, No. (% of subgroup)	p-value
Total No. of Women Enrolled	500	190 (38.0)	
Age groups, y, No. (%)			p <= 1.0
18–24	63 (12.6)	16 (25.4)	
25–34	93 (18.6)	36 (38.7)	
35–44	115 (23.0)	45 (39.1)	
45–54	155 (31.0)	65 (41.9)	
55–64	71 (14.2)	26 (36.6)	
65+	3 (0.6)	2 (66.7)	
Parity, No. (%)			p <= 0.05 ***
Nulliparous	83 (16.6)	40 (48.2)	
Parous	417 (83.4)	150 (36.0)	
Age at Menarche, years, No. (%)			p <= 1.0
<=12	241 (48.2)	90 (37.3)	
13–14	193 (38.6)	77 (39.9)	
>=15	61 (12.2)	21 (34.4)	
Missing	5 (1.0)	2 (40)	
Ethnicity, No. (%)			p <= 1.0
Caucasian	445 (89.0)	173 (38.9)	
Non-Caucasian*	55 (11.0)	17 (30.9)	
1^st ^Degree Relatives with breast cancer, No. (%)			p <= 0.1
No	429 (85.8)	156 (36.4)	
Yes, 1	67 (13.4)	31 (46.3)	
Yes, >=2	4 (0.8)	3 (75.0)	
History of breast biopsy, No. (%)			p <= 1.0
Yes**	56 (11.2)	22 (39.3)	
Menstrual status, No. (%)			p <= 1.0
Pre-Menopausal	358 (71.6)	137 (38.3)	
Menopausal	142 (28.4)	53 (37.3)	
Lactation history, No. (%)			p <= 0.1
Never lactated	268 (53.6)	92 (48.4)	
History of lactation	232 (46.4)	98 (51.6)	

* 47 total African American, 1 Asian, and 7 Hispanic. **type of biopsy not reported *** If only pre-menopausal women are included in the analysis of NAF production vs. parity there is no significant difference (p <= 1.0)

### Nipple aspirate fluid analysis

**Figure 3:**
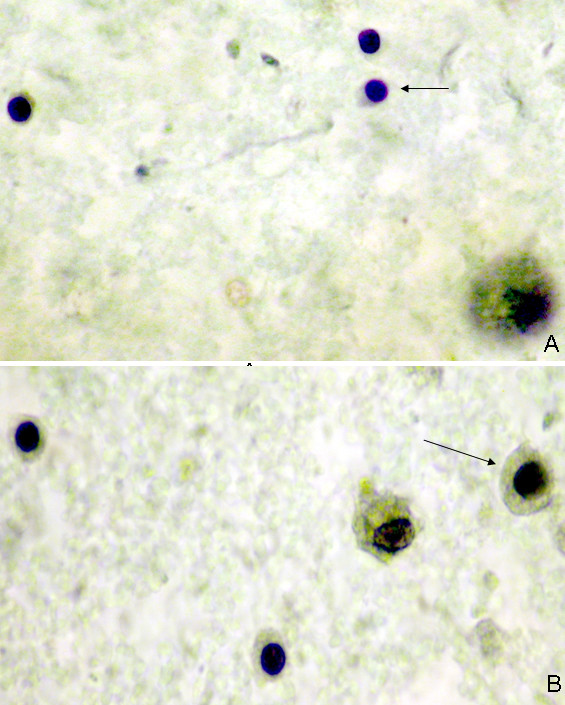
A-B. Category I. Benign (non-hyperplastic) ductal epithelial cells. The breast ductal epithelial cells are single, small, and uniform (arrow-A). Foam cells are a frequent finding (arrow-B). Apocrine metaplastic cells are sometimes identified. Pap 100X.

**Figure 4:**
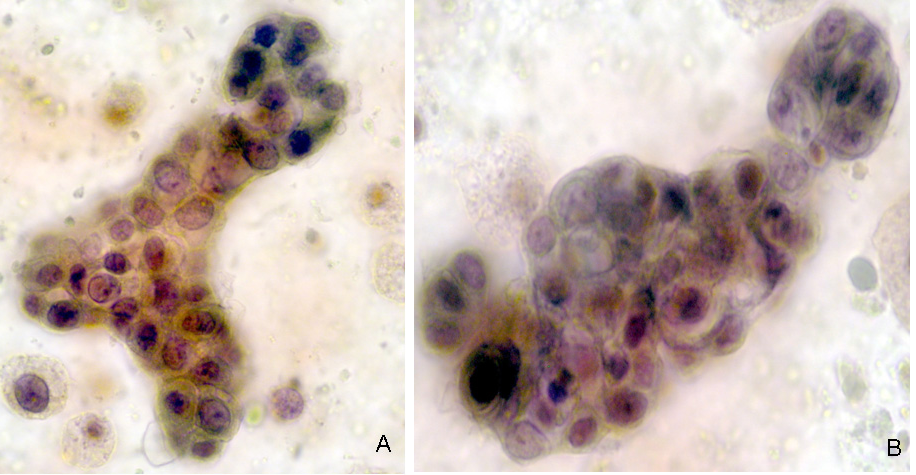
A-B. Category II. Benign hyperplasia. The cells are distributed mainly in cohesive groups of 10–50 cells. Minimal cytologic changes are seen including slight cell and nuclear enlargement. The nuclear chromatin is finely granular and evenly distributed and small regular nucleoli are sometimes present. Pap 100X.

**Figure 5:**
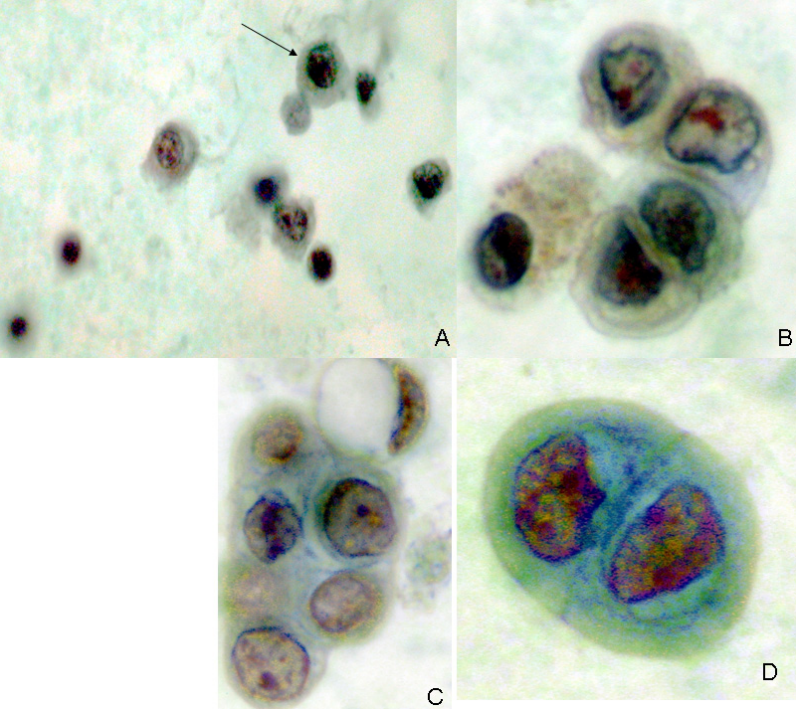
A-D. Category III. Atypical hyperplasia. NAF from a 65-year-old Caucasian woman with a family history of breast cancer and an elevated Gail index of 3.0%. NAF analysis reveals moderate to severe cytologic abnormalities including distinct nuclear enlargement, increasing nuclear to cytoplasmic ratio, irregular nuclear borders, and nuclear variation. The chromatin is coarsely granular and there are prominent chromocenters. While the cells are distributed mainly in groups with occasional papillary formations, there are also increased numbers of single atypical cells (arrow-A). After the Category III findings, a ductal biopsy was performed that was found to be benign. A breast biopsy six months later showed DCIS. Pap 100X.

**Figure 6:**
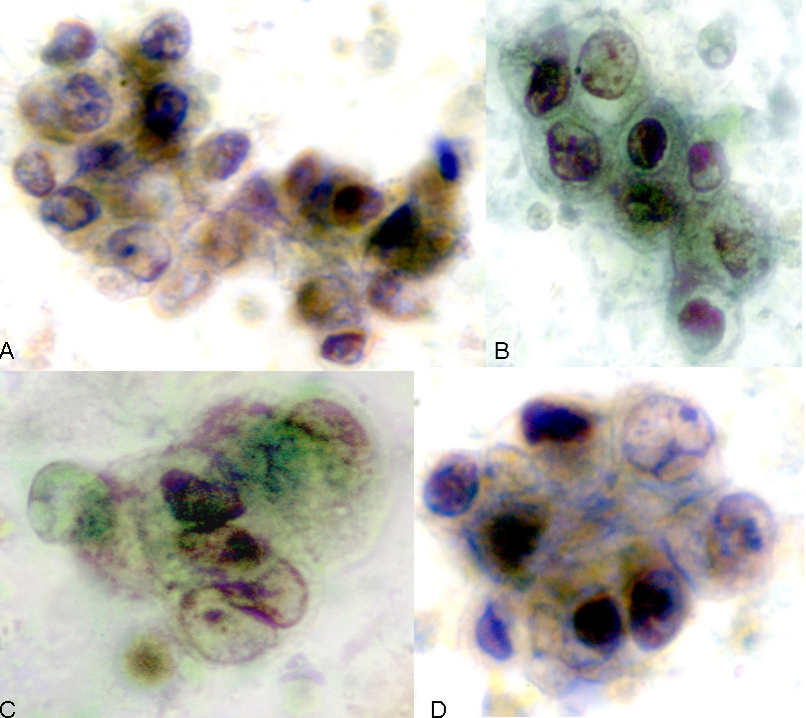
A-D. Category III. Atypical hyperplasia. NAF from a 45-year-old Caucasian woman with a Gail index of 1.1% and no significant medical history. NAF analysis reveals moderate to severe cytologic abnormalities including distinct nuclear enlargement, increasing nuclear to cytoplasmic ratio, irregular nuclear borders, and nuclear variation. The chromatin is coarsely granular and there are prominent chromocenters. Follow-up bilateral biopsies showed LCIS in the right breast, and hyperplastic changes in the left. The changes depicted in these micrographs are not specific for either of those entities and may have originated from other areas of atypia (e.g., DCIS) that was not sampled by the biopsies. Pap 100X.

Three of the 190 specimens collected from the fluid producers had a container leak during specimen transport and therefore could not be analyzed, with the remaining 187 available for evaluation. The final cytology results are summarized in Table 3. Fifty percent (93/187) of the NAF samples were classified as Category 0, 38% (71/187) Category I (Figure 3), 10% (18/187) Category II (Figure 4), and 3% (5/187) Category III (Figures 5 and 6). No Category IV (unequivocal malignancy) samples were identified. Statistical analysis whereby all patients 55+ are combined into one group in order to strengthen the raw numbers showed there was no significant difference between age groups or cytologic categories (p = 0.27).

**Table 3:** Nipple aspirate fluid (NAF) cytologic findings

Cytologic diagnosis	No. of women/Total No. fluid producers (%)	18–24 yr, No. (%)	25–34 yr, No. (%)	35–44 yr, No. (%)	45–54 yr, No. (%)	55–64, No. (%)	65 + yrs, No. (%)
Unsatisfactory (Category 0)	93/187 (49.7)	9/93 (9.7)	14/93 (15.1)	22/93 (23.7)	32/93 (34.4)	15/93 (16.1)	1/93 (1.1)
Benign (Category I)	71/187 (38.0)	5/71 (7.0)	15/71 (21.1)	21/71 (29.6)	25/71 (35.2)	5/71 (7.0)	0
Hyperplasia (Category II)	18/187 (9.6)	1/18 (5.6)	6/18 (33.3)	1/18 (5.6)	5/18 (27.8)	5/18 (27.8)	0
Atypical Hyperplasia (Category III)	5/187 (2.8)	1/5 (20.0)	0	1/5 (20.0)	2/5 (40.0)	0	1/5 (20.0)
Malignancy (Category IV)	0/187 (0.0)	0	0	0	0	0	0
Sample Leak	3/187 (1.6)	0	1	0	1	1	0

### Procedure acceptance and adverse events

A total of 419/500 (84%) women were surveyed for procedure acceptance four to eight weeks after their procedure. The average comfort assessment rating immediate post-procedure was 5.0 on a scale of 1–10 (one being most comfortable) and 4.2 at the four to eight week telephone/mail post-procedure survey (Table 4). The nipple, areola, and breast areas were visually assessed by the study nurse immediately following the procedure. Twenty-six percent of the participants had no observed skin redness after the procedure, 59% mild redness, 14% moderate redness and less than one per cent had severe redness reported. No major adverse events were reported. Two participants chose to discontinue the procedure mid-cycle due to discomfort and there were five reported minor events including bleeding or small surface lacerations. These were treated with topical ointment, observation, Keflex for one suspected mild mastitis, and Mycolog for candiasis noted in one participant. All resolved without further intervention. Eighty-three percent of the participants reported that they would have the HALO procedure again and 88% said they would recommend the procedure to others.

**Table 4:** Patient acceptance and adverse events

	Initial (n = 500)	Post-procedure survey (n = 419)
HALO comfort assessment (scale 0–10)	5.0	4.2
Redness post HALO	No. (%)	No. (%)
None	130 (26.0)	0
Mild	297 (59.4)	0
Moderate	71 (14.2)	0
Severe	2 (0.4)	0
Would recommend HALO to others	N/A	367 (87.6)
Would choose HALO again	N/A	349 (83.3)

### Nipple aspirate fluid and Gail score

Gail 5 year risk profiles were obtained for the participants over the age of 35. Overall, no statistical difference was seen with regards to fluid production and calculated Gail profile result (p = 0.2). Comparison of Gail risk (>1.7% vs. <1.7%) and cytology category results, for the 190 women assessed, showed no significant difference (p = 0.68).

### Post-procedure monitoring

The five women found to have Category III changes were referred for further breast care by their regular physicians. One of these women initially had a benign breast biopsy but was subsequently noted to have ductal carcinoma in situ (DCIS) on a follow-up repeat breast biopsy six months later done as part of an increased surveillance plan as determined by her physician. A second woman had negative bilateral biopsies and the third woman with surgical follow-up had a biopsy that showed lobular carcinoma in situ (LCIS). One of the five Category III women had negative follow-up ultrasound imaging and is being closely followed with mammography every six months. The last of the Category III women, a 24-year-old Caucasian woman with a strong family history of breast cancer in her mother, maternal grandmother and aunt (all diagnosed premenopausally), had negative initial imaging but was subsequently found to have a lump by clinical breast exam. Follow-up ultrasound imaging was again negative. It was recommended that this woman undergo genetic counseling. Follow-up information was not obtained for the Category 0, I or II women and further follow-up of the Category III subjects was not part of this protocol. The Category III women are being followed as high-risk individuals per their physicians' standard protocol outside of this investigation. Only one of these Category III women was identified as being high-risk prior to the NAF collection, using the Gail risk profile calculated at enrollment.

## Discussion

Many studies have shown that finding atypical hyperplasia of the breast ductal epithelium is associated with an increased risk of subsequent development of breast cancer [1-7]. Wrensch and colleagues have observed in a prospective trial that NAF production and NAF atypia in a screening population are associated with an increased risk of breast cancer. Further, cytologic assessment of NAF may modestly improve the discriminatory accuracy of the Gail risk model in a screening population [10]. However, NAF collection requires time and experienced trained personnel. We report prospective cytologic examination of NAF collected from otherwise asymptomatic healthy women obtained in a pilot study using the automated HALO System. We found that it is technically feasible to detect normal and atypical breast ductal epithelial cells using routine cytologic preparation methods and a modified classification system, adapted from King et al [4]. Thirty-eight percent of participants produced a NAF sample and of the samples obtained, 50% had adequate ductal epithelial cells for cytology analysis and five asymptomatic women (5/500, 1%) had Category III changes (atypical hyperplasia). Compared to other studies reporting non-invasive NAF collection (Table 5), the percentage of participants who produced fluid using the HALO collection system falls into the range of these previous manual methods (18%–74%). We observed that 19% of the participants had adequate cellularity (defined in this study as greater than 10 ductal epithelial cells present) which is similar to the few studies that recorded cytology results, although there is wide variability (18%–71%).

**Table 5:** Comparison of non-invasive NAF collection by series

**Series**	**Subject Population**	**Fluid Acquisition Method**	**% Fluid Obtained**	**% samples with >10 ductal epithelial cells present for cytologic assessment**
Papanicolaou et al 1958 [2]	n = 917 asymptomatic women; 19–75 yrs	Manual compression followed by manual breast pump	18%	Specimen cellularity not specifically reported; ~50% of samples "sparsely cellular with no evidence of atypia"
Petrakis et al 1975 [15]	n = 606 healthy volunteers; >18 yrs; Caucasian, Filipina, African American, Mexican, Asian	Manual compression & suction	48%	Findings reported as secretor or non-secretor; cellularity not reported
Sartorius 1977 [3]	n = 203 without breast disease; n = 1503 patients with positive or suspect breast disease	Sartorius syringe – device; manual compression & suction	65% (age 31–50); 30–40% (age <20/>60)	48% of 203 without disease; 54% of women with known or suspected breast disease
Buehring 1979 [16]	n = 1744 self-selected; mostly asymptomatic volunteers; >18 yrs; Caucasian	Sartorius method	49%	36% NAF samples; 18% overall "satisfactory"
Petrakis et al 1981 [17]	n = 3929 volunteers from health fairs; >=18 yrs; Caucasian	Sartorius method	56%	Findings reported as secretor or non-secretor; cellularity not reported
Wynder et al 1981 [18]	n = 244 Finnish volunteers; age 20–69	Sartorius method; repeated up to 10 min	38%	Cytology not assessed
Wynder et al 1985 [19]	n = 990 volunteers; age 30–70 (289 "healthy"'; 548 with benign breast disease; 153 with untreated breast cancer)	Sartorius method; repeated up to 10 min	38–57%	Cytology not assessed
Wrensch et al 1990 [13]	n = 1428 with no history of breast cancer; age 20–74	Sartorius method	37%	Findings reported as secretor or non-secretor; cellularity not reported
Wrensch et al 1992 [8]	n = 2701 Caucasian volunteers; free from breast cancer	Sartorius method	74%	87% NAF samples; 71% overall fluid with satisfactory cytology
Sauter et al 1997 [12]	n = 177 non-Asian subjects including women with history of breast cancer, precancerous mastopathy and invasive cancer	Modified breast pump consisting of syringe attached to endotracheal tube and respiratory humidification adapter	94–99%	96% NAF samples; 53% sufficiently cellular for DNA analysis
HALO Series 2004	n = 500 healthy volunteers; ages 18–65 yrs; asymptomatic, no breast cancer history	Automated five minute cycle (heat, suction, compression)	38%	50% NAF samples; 19% overall produced samples with >10 ductal epithelial cells

Overall, the HALO NAF collection procedure was found to be acceptable by the women studied, rapid, and posed little physical risk. This device has an advantage over other methods of NAF collection in that it is automatic and easy to use, thereby removing most clinician variability. It is also less invasive than other methods of sampling breast epithelium in asymptomatic women, such as ductal lavage and fine needle aspiration.

Criticisms of using NAF to detect cellular abnormalities include the observation that fewer adequately cellular specimens are obtained than with ductal lavage. Dooley et al compared NAF and DL specimens [20] and found that on average a larger number of breast ductal epithelial cells were obtained with DL (13,500) versus nipple aspiration (120) leading to a greater percentage of adequately cellular specimens obtained with DL versus nipple fluid aspiration (78% versus 27% in their study, respectively). In addition, they found abnormal cytologic findings in a greater percentage of DL specimens (24%) than in NAF specimens (9%). In the present study, 38% of the participants produced fluid with the HALO NAF collection system, and of these 50% had specimens that were adequately cellular giving a 19% overall adequacy rate. Even though not all women produce NAF, Wrensch et al [9] showed that women who produce nipple fluid had a slight (1.5 times) increase in the relative risk of breast cancer development and non-producers had a decreased relative risk compared to all fluid producers regardless of final diagnosis. One of Papanicolaou's early studies [1] reported obtaining nipple secretions via breast palpation, massage or a hand-held breast pump, from approximately 50% of the patient population studied; however, this percentage also included women with spontaneous nipple discharge. Secretions were obtained from approximately 19% of asymptomatic women, with pre-menopausal women more likely to produce fluid than post-menopausal women in the Papanicolaou study. Sartorius et al [3] developed a hand held aspiration device, composed of a syringe attached to a small plastic cup placed over the nipple. Their study obtained fluid from approximately 50% of the symptomatic women studied. Wrensch et al [8,9] were able to obtain fluid using a similar device as that described by Sartorius from 40–80% of the women studied, with greater percentages obtained in pre-menopausal women. Krishnamurthy et al [21] obtained fluid from 81% of their study participants; however, these patients had known cancer diagnoses and were under general anesthesia. One pilot study has suggested that administering nasal oxytocin prior to collection can improve the yield of NAF [22].

Despite these limitations, the ease and convenience of this method of obtaining breast ductal epithelial cells might make it a more acceptable option for women who are undergoing NAF collection. As shown in this study, participant acceptance of the procedure was adequate with an average initial comfort assessment rating of 5.0 on a scale of 1–10. Dooley et al [20] reported a lower pain rating for nipple aspiration (8 mm on a 100 mm scale where 0 mm represented "no pain") yet all of the patients in that study underwent either local or general anesthesia. Eighty-three percent of the participants in the current study reported they would have the HALO procedure again and 88% said they would recommend the procedure to others. Since it can be quickly performed in an office setting, patients do not need to be referred to a specialist or scheduled in advance, and can potentially have the test performed at the time of their annual gynecologic exam. The procedure is automated to reduce operator variability and can be performed by non-physician staff.

No prospective studies have been done to date that assess the negative predictive value of ductal cytology in asymptomatic or high risk women. Nipple aspirate fluid assessment is not a diagnostic test for breast cancer. NAF production and cytologic assessment may be used in conjunction with the Gail model for risk prediction and the automated HALO system may facilitate NAF collection in the office setting.

While clinical follow-up care of patients with abnormal breast fluid cytology is not standardized, Shaughnessy et al [23] published an algorithm for management of high-risk women who undergo either nipple aspiration or ductal lavage. Interim management guidelines from the Breast Cancer Risk Assessment Group (BCRAG) [24] also emphasized the need for patients with cytologic abnormalities to undergo a diagnostic work-up to discover potential occult lesion.

NAF specimens have important applications in addition to the cytologic assessments that are described in this paper. A study by Chagpar et al [25] showed that NAF specimens could be tested for the presence of the Thomsen-Friedenreich (TF) antigen, which has been found to be elevated in breast carcinoma. They found a significant difference between healthy breasts and breasts with early-stage carcinoma, even when measured on NAF samples as small as 15 μL. Fackler et al [26] recently reported a new method (quantitative multiplex methylation-specific PCR) that assesses for promoter hypermethylation of DNA in breast ductal epithelial cells, even in samples with as few as 50 cells. This method showed high sensitivity (84%) and specificity (89%) for four genes seen in breast carcinomas. Prostate specific antigen levels in NAF have been shown to decrease with advanced disease stage, larger tumor size, and nodal involvement in women with breast cancer [27]. As additional proteomic, biochemical and genomic biomarkers are identified, testing of NAF samples may become more commonplace.

## Conclusion

The HALO procedure has been shown to be a feasible way to obtain NAF samples for cytologic assessment and it appears to be a safe, rapid, fairly well-tolerated, and non-invasive procedure. While fluid production and adequacy rates may not be as high as reported in manual NAF collection series, there may be advantages to using the HALO System over manual collection techniques. The collection cycle is automated, thus removing any clinician variability and allowing women to be consistently, objectively screened routinely to assess any NAF changes. The system is user-friendly, requires minimal training and can be performed by clinical staff. The system is designed for bilateral, simultaneous collection using heat, compression, and suction combined in a single five minute cycle. Further prospective studies with long-term clinical follow-up are necessary to determine the clinical significance of non-producers vs. producers, insufficient samples and other cytologic categories found on NAF samples collected with the HALO System.

## Competing interests

The author(s) declare that they have no competing interests.

## Authors' contributions

All authors contributing equally to this work. Dr. Proctor is a pathology resident. Dr. Proctor tabulated the final study data and wrote the draft of the manuscript. Ms. Rowe was the cytology data coordinator and research and development scientist. Dr. Bentz was Director of Cytopathology, and responsible for review of study samples. All authors read and approved this manuscript.

## Pre-publication history

The pre-publication history for this paper can be accessed here:


